# Climate change effects on bread wheat phenology and grain quality: A case study in the north of Italy

**DOI:** 10.3389/fpls.2022.936991

**Published:** 2022-08-09

**Authors:** Giovanni Maria Poggi, Iris Aloisi, Simona Corneti, Erika Esposito, Marina Naldi, Jessica Fiori, Stefano Piana, Francesca Ventura

**Affiliations:** ^1^Department of Biological, Geological and Environmental Sciences (BiGeA), Alma Mater Studiorum—University of Bologna, Bologna, Italy; ^2^Department of Agricultural and Food Sciences (DISTAL), Alma Mater Studiorum—University of Bologna, Bologna, Italy; ^3^Scientific Institute for Research, Hospitalization and Healthcare (IRCCS), Institute of Neurological Sciences of Bologna (ISNB), Bologna, Italy; ^4^Department of Pharmacy and Biotechnology (FaBit), Alma Mater Studiorum—University of Bologna, Bologna, Italy; ^5^Department of Chemistry “G. Ciamician”, Alma Mater Studiorum—University of Bologna, Bologna, Italy

**Keywords:** BBCH scale, growing degree days, gluten, gliadins, glutenins, Southern Europe, mediterranean basin

## Abstract

Increasing temperatures, heat waves, and reduction of annual precipitation are all the expressions of climate change (CC), strongly affecting bread wheat (*Triticum aestivum* L.) grain yield in Southern Europe. Being temperature the major driving force of plants’ phenological development, these variations also have effects on wheat phenology, with possible consequences on grain quality, and gluten protein accumulation. Here, through a case study in the Bolognese Plain (North of Italy), we assessed the effects of CC in the area, the impacts on bread wheat phenological development, and the consequences on grain gluten quality. The increasing trend in mean annual air temperature in the area since 1952 was significant, with a breakpoint identified in 1989, rising from 12.7 to 14.1°C, accompanied by the signals of increasing aridity, i.e., increase in water table depth. Bread wheat phenological development was compared in two 15-year periods before and after the breakpoint, i.e., 1952–1966 (past period), and 2006–2020 (present period), the latest characterized by aridity and increased temperatures. A significant shortening of the chronological time necessary to reach the main phenological phases was observed for the present period compared to the past period, finally shortening the whole life cycle. This reduction, as well as the higher temperature regime, affected gluten accumulation during the grain-filling process, as emerged analyzing gluten composition in grain samples of the same variety harvested in the area both before and after the breakpoint in temperature. In particular, the proportion of gluten polymers (i.e., gliadins, high and low molecular weight glutenins, and their ratio) showed a strong and significant correlation with cumulative growing degree days (CGDDs) accumulated during the grain filling. Higher CGDD values during the period, typical of CC in Southern Europe, accounting for higher temperature and faster grain filling, correlated with gliadins, high molecular weight glutenins, and their proportion with low molecular weight glutenins. In summary, herein reported, data might contribute to assessing the effects of CC on wheat phenology and quality, representing a tool for both predictive purposes and decision supporting systems for farmers, as well as can guide future breeding choices for varietal innovation.

## Introduction

Bread wheat (*Triticum aestivum* L.) is one of the most important cereals worldwide, representing a fundamental staple food. In Southern Europe, bread and durum wheat cropping ranks first among cereals for the harvested area (4.98 Mha) and second for production quantity (20.74 Mtonnes), behind maize (FAOSTAT, 2022^1^, visited on 05/01/2022).

This productivity is strongly hampered by climate change (CC), which is threatening food security by the increase in extreme weather events and abiotic stresses (floods, drought, and heat stress), leading to the exasperation of agricultural production losses (Anderson et al., 2020). In general, it has been predicted that a 2.0°C rise in average temperature can lead to a more than 20 to 40% reduction in cereal grain production (Fatima et al., 2020). Specifically, Southern Europe (Mediterranean Europe) experienced a trend of increasing temperature in the last century, with more frequent heat waves and fewer frost days, accompanied by a reduction in annual precipitation (Kovats et al., 2014). The latest projections show that an exacerbation of this trend is expected in the next future, accompanied by more frequent dry spells (Kovats et al., 2014). Climate change-induced warming in Southern Europe has caused a reduction in wheat yield of about 5%, with particular severity in Italy, where temperature increase is accompanied by a decrease in water availability (Moore and Lobell, 2015). Recently, high-resolution impacts of CC on the yield of durum wheat, bread wheat, and maize in Italy were predicted, suggesting different scenarios for those crops relevant to the Italian agricultural economy. In particular, maize and bread wheat will be the most affected crops under the climate change projections, with drastic reductions in yield, estimated between 20 and 43% in maize and 25 and 50% in bread wheat, according to the latitude and to different pedological and crop management features (Mereu et al., 2021).

The identification of threshold temperatures is a very important aspect of CC risk assessment; hence, they have been calculated for the most important crops and recently reviewed, together with the extreme threshold temperatures for the major cereal crops (Luo, 2011; Ramirez-Villegas et al., 2013; Fatima et al., 2020). Moreover, besides threshold temperatures, the impact of temperature variations on plant phenology is to take into account when it comes up to crop yield. In fact, variations in temperature trends have huge consequences on plants’ phenology – defined as the study of the periodically recurring events in plants’ development along with their life cycle (Lieth, 1974, according to Piao et al., 2019) – as the temperature is known to be the major driving force of plants’ phenological development (Chuine and Régnière, 2017). The link between temperature and phenology is expressed in growing degree days (GDDs), calculated starting from air temperature. A growing degree day is a measure of heat accumulated above a specific base temperature (specific for each plant species) during a day (24-period) (Herms, 2004). One degree day is accumulated for each degree that the temperature remains above the threshold typical of the selected plant species, during 24 h; the crop can thus accumulate several GDDs in a day. In fact, plant’s development only occurs between an upper and a lower threshold temperatures. Development stops when the temperature drops below the lower threshold (the base temperature for calculating GDDs) and starts again when it rises above it. In addition, plant’s development stops when the temperature exceeds the upper threshold (Herms, 2004). Years of phenological studies on agricultural crops have made it possible to identify optimum, maximum, and minimum threshold temperatures, as well as how many GDDs need to be accumulated for reaching the various phenological stages, expressed as cumulative growing degree days (CGDDs), even if genotype variations exist (Porter and Gawith, 1999). Climate change has greatly shifted the timing of major phenological events (Piao et al., 2019), and these shifts have been recently predicted in two CC scenarios for Italy, where the spatial distribution of projected changes in the length of the growing cycle for bread wheat indicated earlier maturity for this crops, with a progressively reduced number of days needed to reach the crop maturity moving toward the end of the century (Mereu et al., 2021).

However, the comprehensive analysis of how CC already influenced wheat phenology in this area, and its consequences in terms of grain quality, are still missing. In fact, significant phenological changes can have not only deep impacts on agricultural management and crops performances (Richardson et al., 2013; Piao et al., 2017, 2019), but also alter crop quality in terms of protein, sugars, fiber, and secondary metabolite content (Dusenge et al., 2019; Leisner, 2020). Specifically, grain-filling period, i.e., the developing stage during which nutrients are accumulated as storage molecules in the kernel, going from watery ripe to hard dough, is the most sensitive for wheat grain technological quality (Moldestad et al., 2011; Naeem et al., 2012; Koga et al., 2015). This depends on the fact that gluten proteins, which confer the physical and chemical properties to wheat dough, are accumulated starting from 10 days after anthesis throughout grain filling until the maturation phase at the end of seed development (Shewry, 2019).

Gluten proteins are classified into two groups: the monomeric gliadins, which confer viscosity, and the polymeric glutenins, which contribute to elasticity (Shewry, 2019). Gliadins are further classified into α-, γ-, and ω-gliadins whereas the glutenin polymers consist of two groups, i.e., high molecular weight glutenin subunits (HMW-GSs) and low molecular weight glutenin subunits (LMW-GSs). LMW-GSs have one or two cysteine residues that form inter-molecular disulfide bonds either with HMW-GS or with gliadins, finally forming large three-dimensional networks stabilized by non-covalent forces, particularly hydrogen bonds (Shewry et al., 2009).

Several pieces of evidence demonstrate that gluten composition, quality, and general kernel protein composition, in general, can be severely influenced by growth conditions (Altenbach et al., 2002; Johansson et al., 2013). However, environmental effects on the gluten net and thus final bread-making quality are still not fully understood (Koga et al., 2015). Temperature during grain filling is one of the most important factors that affect gluten quality, which has been known since the early 1990s (Blumenthal et al., 1991, 1993). Since large areas of wheat cultivation experience rising temperatures during the growing season, the effects on wheat quality are well documented and, in general, high temperatures during grain filling are associated with the changes in the composition of gluten proteins (Spiertz et al., 2006; Vida et al., 2014; Koga et al., 2016; Labuschagne et al., 2016 and reviewed in Altenbach, 2012; Wang and Liu, 2021). Often, a decrease in the glutenin to gliadin ratio (Dupont et al., 2006) and an increase in the proportion of large glutenin polymers (HMW-GS/LMW-GS ratio) occur, finally affecting dough strength (Moldestad et al., 2011; Hurkman et al., 2013); however, a strong genotype × environment interaction has been reported, not allowing to state general trends (Vida et al., 2014). These effects are related to the consequences of high temperatures on shortening the grain-filling period, reducing the duration of dry matter accumulation in the seeds, and finally reducing kernel weight (Dupont et al., 2006).

The aim of this study was to assess and quantify the alteration in bread wheat phenological development caused by CC in a key area for its cultivation, such as Southern Europe, and its consequences on grain gluten quality, through a case study in the North of Italy. A precise evaluation of CC impacts in the experimental site was performed, providing punctual meteorological data since 1952. To uncover phenological alterations, bread wheat phenological development in the area in two periods, before and after the breakpoint in temperature increase, was compared, thanks to historical data collections. Moreover, thanks to the availability of modern and ancient grain samples of the same wheat variety, derived from experimental and certification activities carried out in the past, we aimed at verifying in a historical seed collection whether phenological perturbation and higher temperatures regimes caused by CC also produced alterations in gluten composition, which is essential for bread wheat grain quality.

## Materials and methods

### Experimental site

Phenological, meteorological, and grain quality data were analyzed in the Bolognese Plain, located in Emilia-Romagna. Emilia-Romagna is a region in the North of Italy characterized by a subhumid climate with an average annual air temperature equal to 12.8°C and a mean annual precipitation amount of 924 mm (Antolini et al., 2017). CC effects have become evident in the region. In particular, an increasing trend in mean maximum and minimum air temperatures has been observed, accompanied by an increase in heat waves duration and a reduction in winter frost days in the whole region, both in plain and in hill areas (Tomozeiu et al., 2006), in the full manifestation of CC trends for Southern Europe. In particular, the “Climate change mitigation and adaptation strategy for the Emilia-Romagna Region” summary document (Regione Emilia-Romagna, 2018) states that, in the 1991–2016 reference period, the maximum annual temperature recorded an average increase of about 1.5°C compared to the 1961–1990 period (17.8°C compared to 16.3°C). At a seasonal level, there is a greater recorded increase during the summer, with a trend of 0.6°C per decade for maximum temperatures and of 0.3°C per decade for minimum temperatures. The chosen experimental site for phenological and meteorological data evaluated in this study is the locality of Cadriano (44° 33′ 03″ N, 11° 24′ 36″ E), where is located the main agrometeorological station of the Department of Agricultural and Food Sciences (DISTAL) of the University of Bologna, as well as the agro-phenological station of the Department. When it was first installed, the agrometeorological station of the Department was located in Corticella (Lat 44° 32′ 58″ N, 11° 21′ 15″ E) and then moved to Cadriano in 1971. Corticella and Cadriano sites are located in a similar morphological area of the Bolognese Plain, at a distance of 4 km from each other.

### Phenological data

To evaluate CC effects on bread wheat phenology in the Emilia-Romagna region, two 15-year periods have been compared: the first period includes the 15 growing seasons from 1951–1952 to 1965–1966 (past period), and the second one includes 15 growing seasons going from 2005–2006 to 2019–2020 (present period). Phenological data for the past period on four wheat varieties, i.e., San Giorgio, San Pastore, Mara, and Fortunato, were obtained from a bibliography evaluating the performances of these varieties in long-lasting agronomic surveys. The surveys were carried out in open field plots at the experimental farm of the Istituto di Agronomia Generale e Coltivazioni Erbacee of the University of Bologna, that, at that time, was sited in Corticella (BO) (Lat 44° 32′ 58″ N, 11° 21′ 15″ E) (Quagliotti, 1957; Antoniani, 1960, 1971). These experiments were very detailed and accurate, but only lasted for 15 years. This bibliography represents the only available source of information providing phenological data continuously collected on the same varieties in the experimental area in the past. Therefore, for the preclimate change period, we focused on these 15 years of phenological data, collected on the aforementioned four varieties, making a comparison with the latest present. To have homogeneity in the data, we used the same time span of 15 years for the present data. Background information on these four wheat varieties (e.g., frost, rust, and lodging resistance, productivity, thousand kernel weight, hectoliter weight, constitutor name, and pedigree), extensively cropped in the Bolognese Plain in the 20th century, is available in the study by Baldoni (1952).

The same phenological data were obtained for the present period from the phenological bulletin, released weekly by the Department of Agricultural and Food Sciences (DISTAL), University of Bologna. The bulletin publishes the data of the phenological surveys carried out on the cultivar Mieti, in accordance with the Phenagri protocol (Pasquini et al., 2006), at the agro-phenological station experimental plots sited in Cadriano (BO) (44° 33′ 03″’ N, 11° 24′ 36″ E). Mieti is currently used for the bulletin, being well representative of the most varieties used in the area. Phenology was analyzed according to the internationally recognized BBCH scale (Biologische Bundesanstalt, Bundessortenamt, and CHemical industry). The BBCH scale encodes plants’ development stages using a double-digit code, which goes from sowing (00) to harvest (99), thus consisting of 10 principal stages (0–9), with 10 secondary stages (0–9) for each principal one (Meier, 1997). For each variety in each agronomic season, the chronological time, expressed as days after sowing (DAS), and the CGDDs necessary to reach several BBCH stages were calculated. In particular, CGDDs necessary to reach stages 31 (beginning of stem elongation), 40–49 (booting), 50–59 (heading), 61 (beginning of anthesis), and 89 (full ripening) were calculated, using the single-triangle method (Snyder et al., 1999), with a base temperature of 0°C (Steduto et al., 2012).

### Meteorological data

For GDD calculation, to be coupled to phenological data, weather data (daily maximum, minimum, and mean temperature – T_*max*_, T_*min*_, and T_*mean*_) were provided by DISTAL agrometeorological station, nowadays sited in Cadriano, nearby the agro-phenological station where Mieti phenological data are collected for the weekly bulletin. The original position of the station was in Corticella, near the experimental plots of the agronomic surveys on Fortunato, Mara, San Giorgio, and San Pastore, relating to the period 1951/1952–1965/1966, and was then transferred to Cadriano in 1971. Taken together, Corticella and Cadriano daily weather data represent a mechanical historical series that goes from 1 January 1952 to the present. These mechanically measured weather data were used, representing a continuous and uniform historical series from 1952 to today, measured near the plots where the phenological data for both periods were collected. A homogeneity test (Kruskal–Wallis test) was carried out on Corticella and Cadriano data, before and after the station was moved. The test showed homogeneity for both mean temperature and precipitations, as previously reported (Ventura et al., 2002; Matzneller et al., 2010). Hence, the two stations’ data series can be considered as a single, uniform, and homogeneous series. The same weather data for the last quarter of 1951, when Corticella station was not yet installed (necessary to calculate GDDs from the sowing date), were kindly provided by ARPAE Emilia-Romagna and refer to the Bologna IdroGrafico station. Other than T_*mean*_, precipitation data from the same time series and water table depth (measured in Cadriano agrometeorological station every 10 days since 1975) were analyzed to assess the impact of CC in the area where wheat phenological and quality traits were assessed. Past and present 15-year periods were characterized using the Bagnouls and Gaussen climate index (Bagnouls and Gaussen, 1957), coupling temperature and precipitation data, to verify that the two periods considered significantly differ from a climatic point of view. As the definition of Bagnouls – Gaussen climatic index states, the so-called aridity period is identified when the two times-monthly precipitation curve lies below the monthly average temperature curve.

### Grain material for quality assessment

The second aim of this work was to verify whether life cycle shortening and CC caused an alteration in wheat gluten quality. Wheat grains of San Pastore produced in the area of the Emilia-Romagna plain, both before and after the recognized breakpoint in temperature, were analyzed.

We chose to focus on San Pastore, since this is the only one of the four past period varieties that is still currently cropped in the region, for which it was therefore possible to find modern seed samples in the area. Grain samples for the period before the breakpoint have been kindly provided by Laras (Seed Research and Analysis Laboratory of the Department of Agricultural and Food Sciences, University of Bologna, Italy). Laras is an International Seed Testing Association (ISTA) accredited laboratory, where samples of many grain varieties harvested in the last century are still preserved. Local farmers and seed companies have provided modern San Pastore grain samples. Table 1 lists past and modern San Pastore grain samples that we were able to collect for this study and subjected to the analysis. All samples were 5-g weight and stored in proper standard condition by both Laras accredited Laboratory and local farmers and seed companies, with no damaged material.

**TABLE 1:** San Pastore grain samples of pre- and post-temperature breakpoint period used in this study.

Sample number	Year	Provided by	Cultivation site	Temperature breakpoint
1	1951	Laras	Bolognese Plain	pre
2	1955	Laras	Bolognese Plain	pre
3	1955	Laras	Bolognese Plain	pre
4	1955	Laras	Bolognese Plain	pre
5	1961	Laras	Bolognese Plain	Pre
6	1973	Laras	Bolognese Plain	pre
7	2016	SIS s.p.a.	Bolognese Plain	post
8	2020	Casa Vecchia farm	Correggio (Emilia-Romagna Plain)	post
9	2020	Zanarini farm	Bolognese Plain	post

### Grain genetic homogeneity

To assess grain genetic homogeneity, all the San Pastore samples collected were subjected to molecular analysis using simple sequence repeat (SSR) markers. Specifically, 19 Xgwm SSR markers were probed, chosen among those developed on the Chinese Spring variety (Röder et al., 1998). SSR markers used were chosen randomly across the entire genome and are freely available in the GrainGenes database^2^. Genomic DNA was extracted from seeds using the CTAB method (Murray and Thompson, 1980), and polymerase chain reaction (PCR) (according to amplification conditions specified in GrainGenes marker report) and subsequent gel electrophoresis in 2% agarose gel were performed.

### Gluten fraction extraction, sodium dodecyl sulfate–polyacrylamide gel electrophoresis, and analysis by liquid chromatography – high-resolution mass spectrometry

#### Chemicals and reagents

Chymotrypsin, hydrochloric acid, dithiothreitol (DTT), iodoacetamide (IAA), and ammonium bicarbonate were provided by Merck (Darmstadt, Germany). Ultrahigh-performance liquid chromatography-mass spectrometry (UHPLC-MS)-grade acetonitrile and water were provided by VWR chemicals (Radnor, PA, United States). LC-MS-grade formic acid (FA) was provided by Carlo Erba Reagents S.r.l. (Milan, Italy). RC membrane 0.2-μm 4-mm syringe filters were provided by Phenomenex (Torrance, CA, United States).

#### Sample preparation

Endosperm storage proteins were extracted according to the study by De Santis et al. (2020) with minor modifications. Briefly, 100 mg of flour was suspended in three times 0.4 ml of 0.1 M KCl buffer (pH 7.8) and centrifuged to remove soluble proteins which were discarded. The KCl-insoluble fraction was suspended in isopropanol solution (50% v/v) and centrifuged for 10 min at 4,500 *g* and gliadins were collected. Glutenins were extracted from the pellet by extraction solution (isopropanol 50% v/v, 1% DTT), after centrifugation at 10,000 *g* for 10 min (room temperature).

#### Sodium dodecyl sulfate – polyacrylamide gel electrophoresis and gel staining

Electrophoretic runs were performed according to the study by Ruiz et al. (2016) with minor modifications. Proteins (40 mg lane^–1^) were separated by sodium dodecyl sulfate–polyacrylamide gel electrophoresis (SDS-PAGE) using The Mini Protean TETRA apparatus (Bio-Rad Laboratories, Italy). The molecular mass standard was the Neobiotech (Nanterre, France) Elite Prestained Protein Ladder (6.5–270 kDa). Samples were dried (RapidVap Evaporator Labconco) under vacuum and resuspended in 30 ml MilliQ water, and thus, sample buffer was added [final concentrations 2% SDS, 10% glycerol, 200 mM 2-mercaptoethanol, and 0.01 mg/ml bromophenol blue in 63 mM Tris-HCl (pH 6.8)]. Samples were boiled for 5 min prior to loading. Gels were fixed at room temperature in methanol/glacial acetic acid/water (50/5/45, v/v) for 20 min and then in 50% methanol for 10 min. After fixing, gels were stained with Bio-Safe Coomassie blue (Bio-Rad, Milan, Italy) as described in the instruction protocol.

Gliadins were subdivided into two classes (ω- and α-, γ-) based on their molecular weight, whereas glutenins were subdivided into HMW-GS and LMW-GS (De Santis et al., 2017). Protein profiles were analyzed using the Azurespot Pro software (Azure Biosystems, Dublin, CA, United States).

#### Liquid chromatography–high-resolution mass spectrometry analysis

Extracted protein samples were dried (RapidVap Evaporator Labconco) under vacuum and resuspended with an appropriate amount of 50 mM ammonium bicarbonate buffer pH 7.8 to obtain a final protein concentration of 1.3 mg/ml for all samples. About 85 μl of protein solution was incubated with 5 μl of DTT (100 mM in ammonium bicarbonate buffer 50 mM buffer pH 7.8) at 56°C for 30 min under gentle shaking. Afterward, 10 μl of IAA (55 mM in ammonium bicarbonate buffer 50 mM, pH 7.8) was added. Samples were incubated for 45 min in the dark at room temperature under gentle shaking. About 2 μl of chymotrypsin (1 μg/μl in 2 mM HCl) was added to obtain the final enzyme/protein ratio of 1:55 and samples were digested overnight at 30°C under shaking. Digestion was stopped by adding 2 μl of a 10% aqueous solution of formic acid and samples were 10-folds diluted with 50 mM ammonium bicarbonate buffer pH 7.8. Samples were finally filtered with RC membrane 0.2-μm 4-mm syringe filters, and 5 μl of filtrate was injected for liquid chromatography – high-resolution mass spectrometry (LC-HRMS) analysis.

Liquid chromatography–high-resolution mass spectrometry analysis was performed on an Eksigent M5 MicroLC system (Sciex, Concord, ON, Canada) coupled to a TripleTOF 6600 + mass spectrometer with OptiFlow Turbo V Ion Source (Sciex, Concord, ON, Canada). About 5 μl (5 μg of peptides) from each sample was loaded onto a Kinetex XB C18 (50 mm × 0.5 mm I.D., 2.6 μm 100 Å) column thermostated at 35°C. Mobile phases A consisted of 0.1% FA in water and mobile phases B in 0.1% FA in acetonitrile. Chromatographic separation was achieved over a 25-min linear binary gradient with a constant flow rate of 20 μl/min developed as follows: 0–15 min, 5–40% eluent B; 15–18 min, 40–65% eluent B; 18–20 min, 65–95% eluent B; 20–23 min, 95% eluent B; 23–23.5 min, 95–5% eluent B; 23.5–25 min, 5% eluent B; equilibration time 3 min. The IonSpray voltage (ISV) was set at 5000 V. The curtain gas supply pressure (CUR) was set at 30 PSI; nebulizer and heater gas pressures were set at 30 and 40 PSI, respectively. The ion spray probe temperature was set at 300°C. Declustering potential was 80 V. Analyses were carried out using rolling collision energy.

Samples were analyzed in information-dependent acquisition (IDA) mode followed by a second run in data-independent acquisition mode (SWATH-MS: Sequential Window Acquisition of All Theoretical Mass Spectra). Pepcal Mix (Sciex, Concord, ON, Canada) was used to assure steady MS calibration during the whole analysis time frame.

Obtained data files were processed using PeakView, ProteinPilot, and MarkerView (Sciex, Concord, ON, Canada). LC-MS/MS data were analyzed by searching the “Triticum Aestivum” UniProt database (2021-12-07) allowing only three missed cleavages. “Biological modifications” function was selected as fixed modification. Values of score, matches, and % coverage were retrieved from the obtained report and compared. Peak areas were automatically calculated in SWATH-MS MicroApp using PeakView to compare protein composition among samples and perform correlation analysis with meteorological parameters.

### Statistical analysis

Statistical analysis was performed in R statistical environment (R version 4.0.3^3^, open source). Specifically, R packages used were Kendall, strucchange, stats, lawstat, pgirmess, ggplot2, and ggpubr.

To assess the impact of CC in the area, daily T_*mean*_ data were subjected to analysis for the identification of an increasing trend (Mann-Kendall test) as well as of breakpoints, to verify that the two 15-year periods considered belonged to two different climates in terms of temperature (Dawood, 2017). To assess whether CC impact also includes aridity signals, i.e., an increase in water table depth, the data of the historical series were subjected to Mann-Kendall test, to verify the possible existence of a trend and its statistical significance (Dawood, 2017).

To be sure that any differences found between the varieties for the achievement of BBCH stages 31, 40–49, 50–59, 61, and 89, in terms of chronological time from sowing (DAS), were not due to a different precocity, the corresponding 15 years of CGDDs data for each variety were subjected to statistical comparison. Normal data distribution has been verified by the Shapiro–Wilk test, and when not met, non-parametric Kruskal–Wallis test was used (data homoscedasticity verified by Levene’s test before use), followed by the related *post hoc* to separate the means (Kruskal and Wallis, 1952; Levene, 1961; Shapiro and Wilk, 1965).

To statistically verify the significance of the difference between past period and present period in terms of DAS for the achievement of the considered BBCH stage, the average values of the four varieties of past period over the 15 years were compared with the 15 years values of Mieti, using *t*-test. Wilcoxon rank-sum test was performed when data did not satisfy normal data distribution, verified by Shapiro–Wilk test (Bridge and Sawilowsky, 1999).

To assess the potential impact of CC on wheat gluten quality, grain quality traits investigated, obtained by LC-HRMS analysis, were tested for their correlation with CGDDs accumulated in the period 1 May–15 June, when wheat grain filling typically occurs in the area. CGDDs calculation was performed starting from Corticella and Cadriano daily data (representative of Bolognese Plain). In the 2 years, 1955 and 2020, we have more than one specimen (three and two, respectively): in both cases, the average year value was used. Pearson’s *r* was applied when normal distribution was met (verified by the Shapiro–Wilk test). When not met, Spearman’s *r* was used (de Winter et al., 2016).

## Results

### Climate change in the Bolognese Plain

**FIGURE 1:**
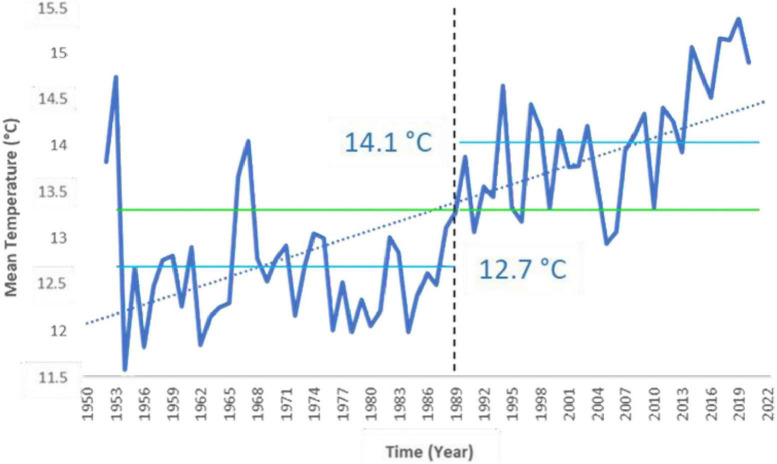
University of Bologna agrometeorological station annual mean air temperature and its increasing trend (dashed blue line). The trend was significant with 95% confidence. A breakpoint in annual mean air temperature was found in 1989. The two labeled lines represent the average temperature for the periods before (T_*mean*_ = 12.7°C) and after (T_*mean*_ = 14.1°C) the breakpoint, respectively.

Analyzing the time series of annual mean air temperature (T_*mean*_, calculated from daily data) of Corticella and Cadriano agrometeorological stations (representative of the area of the Bolognese Plain) from 1952 to 2020, a significant increasing trend has emerged from the Mann-Kendall test (τ = 0.538, *p*-value < 0.05), with a breakpoint found in 1989 (Figure 1). Specifically, mean temperature before and after the breakpoint was, respectively, 12.7 and 14.1°C (Figure 1), with an increase in mean temperature of 1.4°C. Matzneller et al. (2010) already performed this analysis for the period 1952–2007, finding a relevant increasing trend in the T_*mean*_ (of about 1.2°C), with a breakpoint in 1987. Here, we extended that analysis to 2020. Therefore, the selected past period and present period resulted to belong to two different climatic periods for the Bolognese Plain.

**FIGURE 2:**
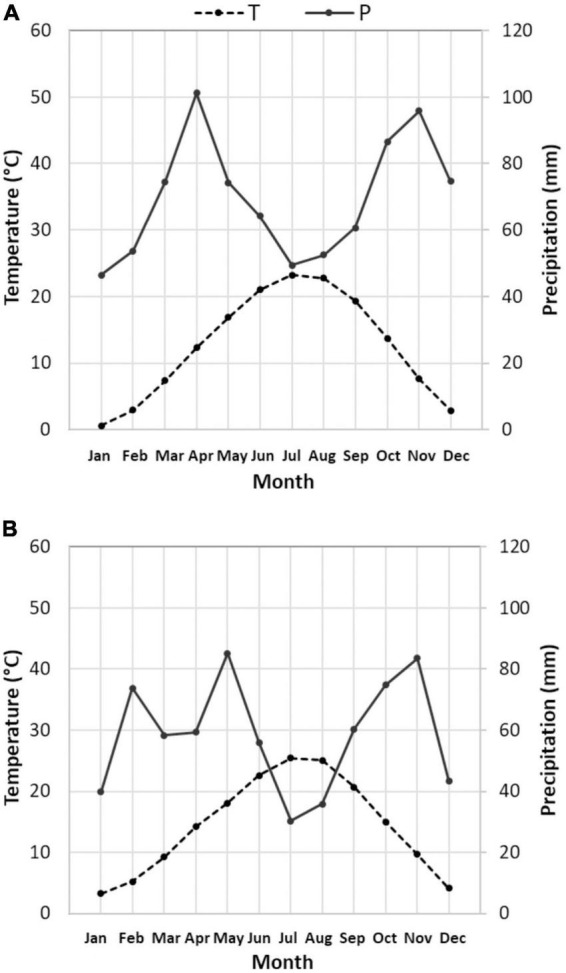
Climatic characterization (Bagnouls–Gaussen diagram) for past **(A)** and present **(B)** period: diagrams are both based on 15 years averaged data, gathered by Corticella-Cadriano continuous and uniform daily mechanical weather data historical series, representative of the Bolognese Plain. Please note that in the Bagnouls and Gaussen representation, the precipitation (P) axis doubles the temperature (T) axis. In this condition, the aridity period is defined as that during which the precipitation line is below the temperature line.

**FIGURE 3:**
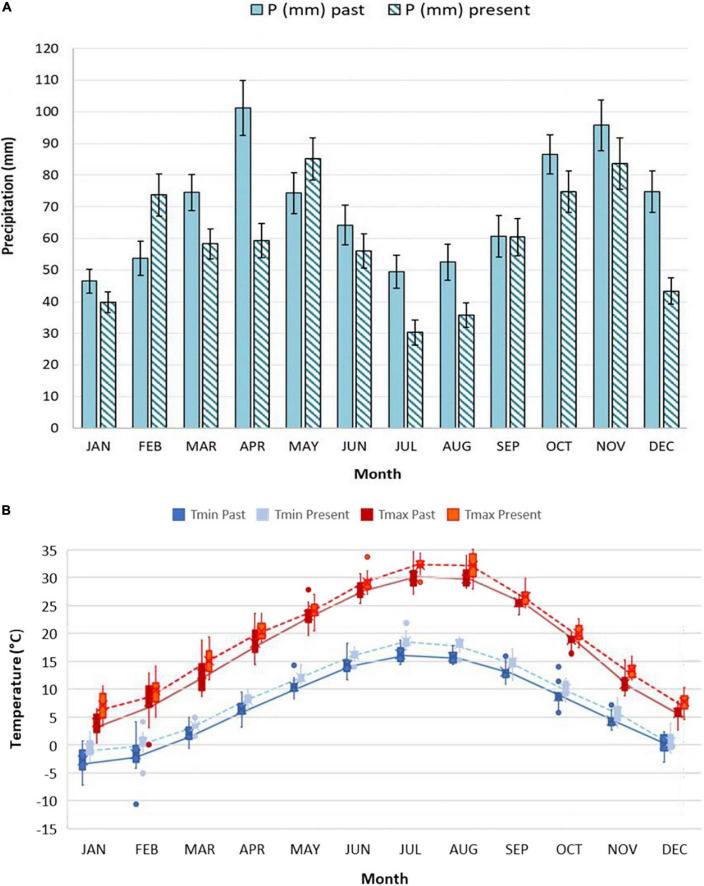
Average monthly precipitation **(A)** and mean monthly T_*max*_ and T_*min*_ **(B)** for past and present period. Figures are both based on 15 years averaged data, gathered by Corticella-Cadriano continuous and uniform daily mechanical weather data historical series, in the Experimental Farm of the University of Bologna. Boxplots for monthly temperatures data are presented: the box goes from first to the third quartile. The line through the box represents the median. The whiskers go from each quartile respectively to the minimum and maximum value. Individual points outside the ends of the whiskers are outliers.

Climatic characterization of past period (Figure 2A) and present period (Figure 2B) is reported, through the Bagnouls and Gaussen diagram. Diagrams represent 15 years of averaged data, obtained starting from the daily weather data of the aforementioned mechanical time series. As the definition of the Bagnouls and Gaussen climatic index states, the so-called aridity period is identified when the two times-monthly precipitation curve lies below the monthly average temperature curve. Present period showed an evident aridity period, which was completely absent in the past, during the months of June till August. In total, the average annual precipitation dropped from 834 mm in the past period to 701 mm in the present period, but the inter-annual variability is so pronounced as to mask any trend in annual rainfall (Pavan et al., 2019). Looking at the monthly data (Figure 3A) and comparing the past with the present period, it is clear that there is a change in the distribution of precipitation, with large decreases in water supplies in April (which was the wettest month) and December. The change in the aridity is also due to the increase in annual T_*mean*_ in the two 15-year periods, from 12.6 to 14.4°C. Changes in T_*max*_ were recorded mostly during the months from January to April, as well as July and August (>2°C on average). January and February, as well as July and August, also showed the highest increases in T_*min*_ (>2°C on average) (Figure 3B).

**FIGURE 4:**
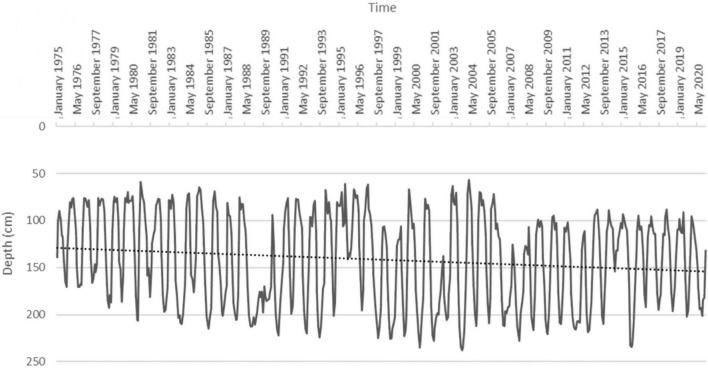
Water table depth in Cadriano from 1975 to 2020. The dashed line represents the increasing depth trend (significant to the Mann-Kendall test).

Analyzing the historical series of water table data, we observed a trend of progressive increase in its depth, significant to the Mann-Kendall test (τ = 0.113, *p*-value < 0.05) (Figure 4). As an annual average, mean water table depth increased from about 130 cm in the 1970s to about 155 cm in the present time.

### Cumulative growing degree days and days after sowing

**FIGURE 5:**
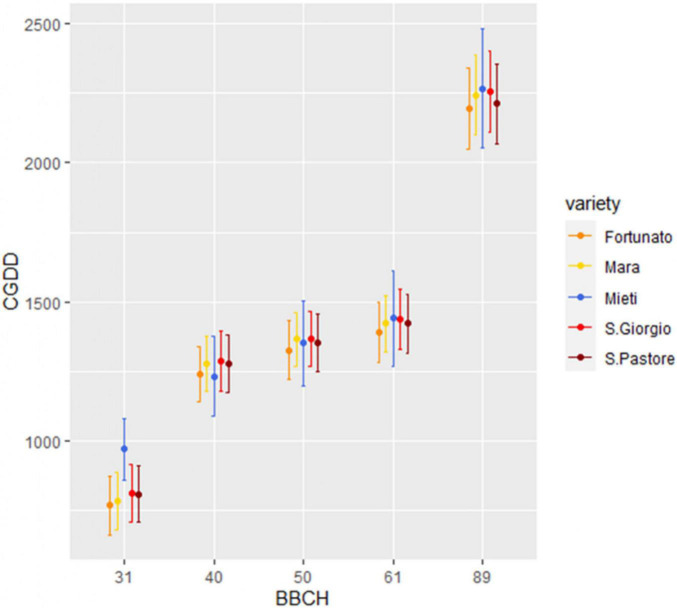
Cumulative growing degree days (CGDDs) necessary to reach the selected BBCH stages, for each variety. Dots represent the mean of 15 agronomic seasons, and upper and lower bars represent one standard deviation. San Pastore, Mara, Fortunato, and San Giorgio belong to the past period, Mieti to the present.

Cumulative growing degree day values necessary to achieve BBCH stages 31, 40–49, 50–59, 61, and 89 in 15 agronomic seasons were calculated using the single-triangle method for five varieties: San Pastore, Mara, Fortunato, and San Giorgio for the period 1951/52–1965/66 and Mieti for the period 2005/06–2019/20. The Kruskal–Wallis test was applied to the data. The test showed a significant difference for phase 31 (*p*-value < 0.05), while it did not show any statistically significant difference for stages 40–49, 50–59, 61, and 89 (*p*-value > 0.05). Dunn’s *post hoc* rank-sum comparison was performed to separate means for the BBCH 31 stage. *Post hoc* test confirmed that Mieti showed a significantly higher mean thermal threshold for reaching stage 31 (971 ± 111 CGDDs), compared to the other four varieties. All the five varieties considered, therefore, showed, starting from the booting phase, the same requirements in terms of thermal thresholds for reaching the phenophases, until the end of their cycle, as shown in Figure 5.

**FIGURE 6:**
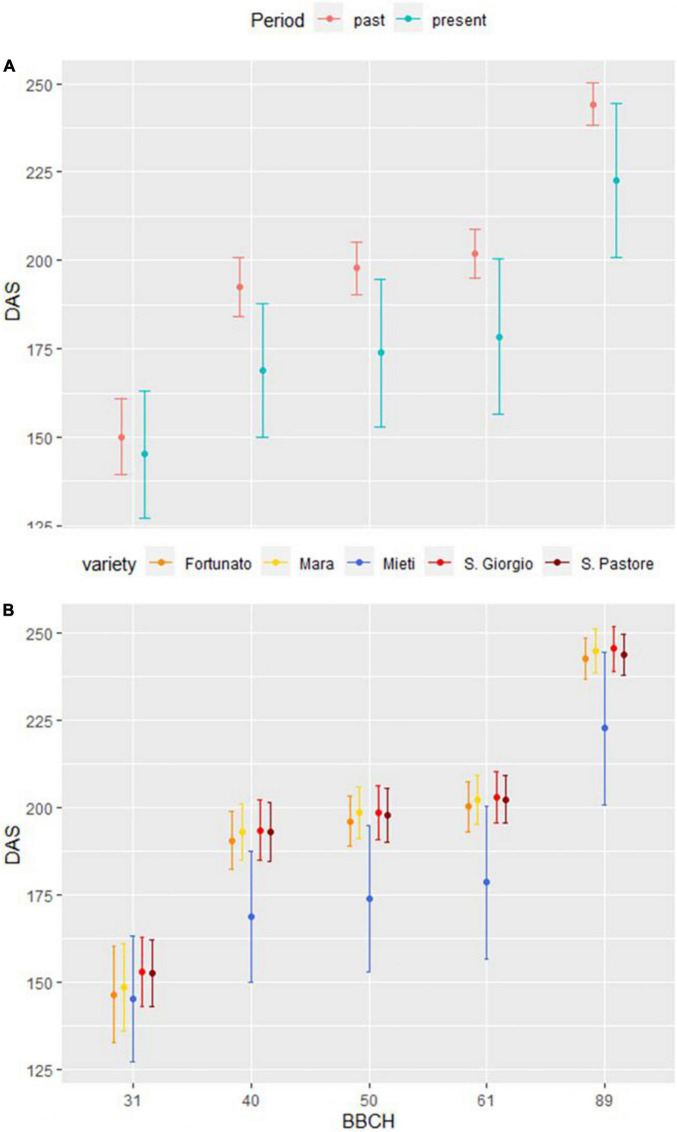
DAS necessary to reach 31, 40–49, 50–59, 61, and 89 BBCH stages in past period (mean of 4 varieties) and present period (Mieti) **(A)**. Data for each variety are also presented **(B)**. Dots represent the mean of 15 agronomic seasons, and upper and lower bars represent one standard deviation.

To verify whether wheat life cycle has significantly shortened compared to the past, especially the generative period going from flowering to ripening, DAS between the average values of the four past period varieties and the values of the present period variety Mieti in 15 growing seasons were compared. For BBCH stage 31, a *t*-test was performed, and no significant difference emerged between the average of the four varieties of the past period and Mieti (present period) (*p*-value > 0.05). As regards BBCH stages 40–49, 50–59, 61, and 89, on the other hand, the Wilcoxon rank-sum test was applied, and a significant shortening of the chronological time necessary to reach the aforementioned phenophases from the sowing date was observed for the present period (*p*-value < 0.05). The average life cycle length in the past period was 244 ± 6 days, compared to the average of 223 ± 22 days in the present period. Figure 6 shows the DAS necessary to reach 31, 40–49, 50–59, 61, and 89 BBCH stage in past period (mean of four varieties) and present period (Mieti) (6A), as well as data for each variety (6B).

### Seed storage protein composition

**FIGURE 7:**
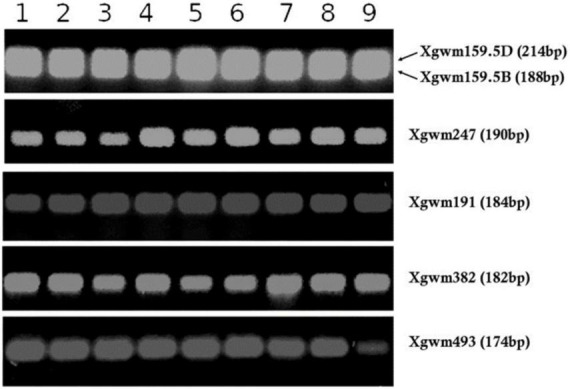
SSR markers profiling (i.e., Xgwm159.5D, Xgwm159.5B, Xgwm247, Xgwm191, Xgwm382, and Xgwm493) for all San Pastore grain samples under analysis. The number reported above corresponds to the sample number exposed in Table 1.

Before protein extraction and analysis in LC-MS, seed samples were examined for genetic homogeneity by molecular analysis using SSR markers to assure that all the samples belonged to the San Pastore variety. All the samples examined were highly homogeneous for the profile of the used markers (Figure 7 and Supplementary Table 1), excluding any genotype involvement in protein composition.

**FIGURE 8:**
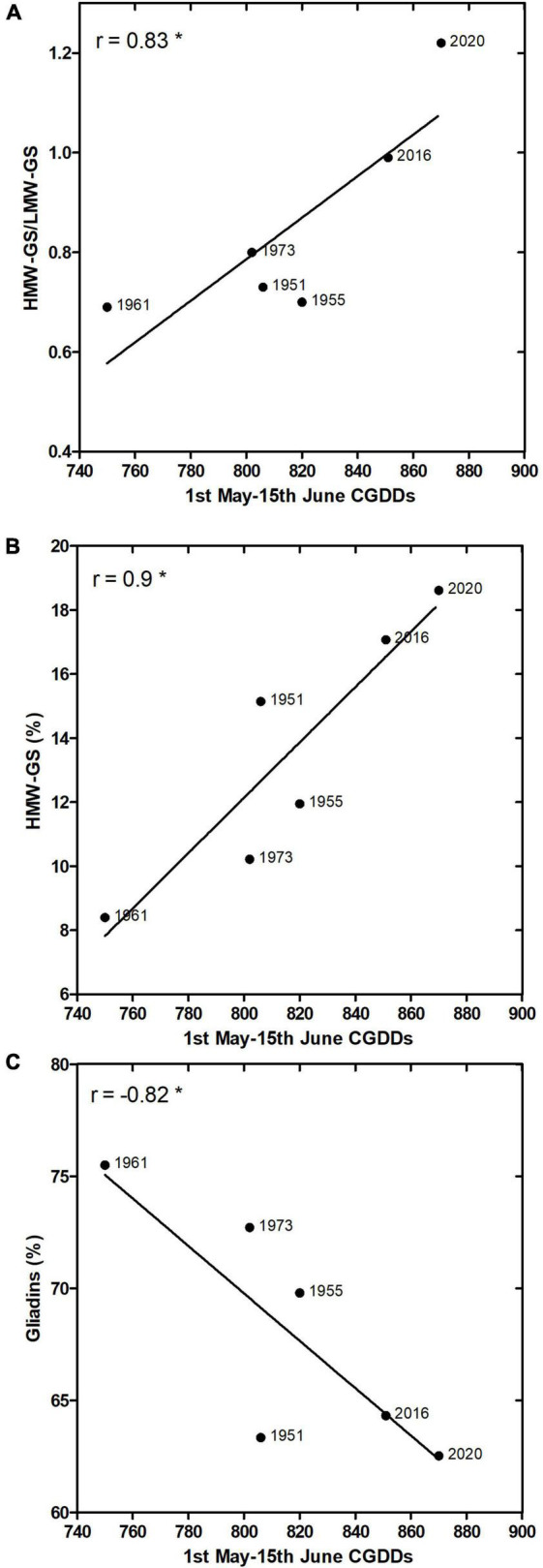
1 May–15 June CGDDs Pearson’s *r* correlation with HMW-GS/LMW-GS ratio **(A)**, HMW-GS (%) **(B)**, and gliadins (%) **(C)**. For 1955 and 2020, average year values are presented. *Means significant at *p* = 0.05 level.

Seed samples were homogenous in total protein content, which was on average 13.1 ± 1.2%, with maximum in the sample of 1955 (14.9%) and minimum in that one of 1973 (11.2%). The gliadin and glutenin fractions accounted together 82–86% of the total protein amount, with maximum in the sample of 1955 (86.07%) and minimum in that one of 2020 (79.55%). Proteins in the gliadin and glutenin fractions were analyzed by LC-MS, identifying 41 gliadins and 22 glutenins (Supplementary Table 2). Gliadins generally accounted for between 66 and 75% in the analyzed samples, whereas glutenins between 27 and 37%. The predominant proteins identified belonged to α-gliadin (23 peaks), followed by γ-gliadins (12 peaks) and ω-gliadins (6 peaks). In total, eight identified sequences corresponded to HMW-GS and 14 to LMW-GS. HMW-GS accounted for between 8 and 18% of the gluten proteins whereas LMW-GS accounted for between 12 and 17% (Table 2). General trends were visible also in SDS-PAGE, e.g., an increase in crude protein and glutenin content, mostly HMW-GS, during the decades under examination. Differently, gliadins showed a decreasing trend (Supplementary Figure 1). Protein fraction distribution was further investigated for correlation with the number of CGDDs accumulated in the period 1 May–15 June, during which wheat grain filling occurs in the region, for each year in analysis. Strong and significant (*p*-value < 0.05) Pearson’s *r* correlation resulted between CGDDs and HMW-GS/LMW-GS ratio (*r* = 0.83) (Figure 8A), HMW-GS (%) (*r* = 0.9) (Figure 8B), and gliadins (%) (*r* = −0.82) (Figure 8C). These traits showed a significant correlation, despite the limited number of samples.

**TABLE 2:** Amount of crude protein (mg/g flour), protein distribution among fractions (%), and ratio between gliadins/glutenins and HMW-GS/LMW-GS in wheat flours derived from kernels harvested in different years.

Year of seed sample	Crude protein (mg/g flour)	Monomeric (Gliadins) (%)	Polymeric (Glutenins) (%)	Monomeric/polymeric	α, γ-gliadin (%)	ω-gliadin (%)	HMW-GS (%)	LMW-GS (%)	Ratio HMW-GS/LMW-GS
1951	130	63.64	36.36	1.73	60.29	3.84	15.14	20.73	0.73
1955	112.50	80.12	19.88	4.03	80.89	2.62	6.13	10.36	0.59
1955	141.20	67.65	32.35	2.09	59.82	10.56	10.67	18.96	0.56
1955	149.5	61.61	38.39	1.6	55.12	5.43	19.04	20.41	0.93
1961	136.20	75.50	24.50	3.08	74.88	4.47	8.40	12.26	0.69
1973	111.60	72.71	27.29	2.66	75.06	2.02	10.22	12.71	0.80
2016	146.10	64.32	35.68	1.80	60.49	5.23	17.07	17.21	0.99
2020	162.80	57.15	42.85	1.33	53.31	4.65	18.90	23.15	0.82
2020	123.4	67.9	32,1	2.12	65.8	4.65	18.32	11.23	1.63
Pearson’s *r* with 1st May–15th June CGDDs	0.44	−0.82*	0.29	−0.5		0.28	0.9*	0.53	0.83*
Spearman’s *r* with 1st May–15th June CGDDs					−0.77				

Pearson’s or Spearman’s *r* correlation value with 1 May–15 June CGDDs are reported (for 1955 and 2020, average year values were used).

*Significant at 0.05 level.

## Discussion

Temperatures have steadily increased since the 1950s in the area of the Bolognese Plain. This trend from 1 January 1952 up to today was significant to the Mann-Kendall test, with a breakpoint in 1989. Therefore, our analysis extended up to 31 December 2020 confirmed what Matzneller et al. found, up to 2007, about the presence of a clear and significant increasing trend in annual mean air temperature. Despite having added 13 years of data to what has already been analyzed (Matzneller et al., 2010), the breakpoint has moved only by 2 years, from 1987 to 1989, demonstrating appreciable robustness of the method used. The increase in mean annual temperature since 1952 was 1.4°C (considering an annual mean temperature of 12.7 and 14.1°C, respectively, before and after the break point). It should be noted that this high value of temperature increase, caused by climate change, refers to a single site and therefore shows an understandable deviation from average regional data. Climate change in the area also affected the water table, which showed a trend of increasing depth since 1975, significant to Mann-Kendall test, demonstrating that the temperature increase was accompanied by the signals of increasing aridity in the area.

This case study aimed to verify whether CC has produced considerable effects on bread wheat phenological development. The topic is of particular interest, given the efforts made to analyze and predict phenology in bread wheat by remote sensing (Wang et al., 2008; He et al., 2015; Wu et al., 2019). This approach, however, has been recently called for paying more attention by authors who compared its reliability with that of phenological studies performed by field observation, highlighting significant discrepancies (Chen et al., 2020). Hence, even if the uneven spatial distribution of ground stations limits the potential to explore the spatial distribution of phenological responses to CC (Liu et al., 2017, Piao et al., 2019; Chen et al., 2020), field observation-based analysis remains the only suitable method for analyzing phenological data of the past, one of the goals of this research. Phenological data of two 15 growing seasons, selected before and after the temperature breakpoint, have been compared in terms of CGDDs and DAS necessary to reach BBCH stages 31, 40–49, 50–59, 61, and 89. The climatic characterization of the past and present periods, through the Bagnouls–Gaussen diagram, confirmed that the two 15-year periods examined showed different climatic characteristics. As for CGDDs, the thermal threshold necessary for present period variety to reach phenophase 31 appeared significantly higher than the thresholds required by the varieties of past period. In the literature, there is evidence of a genetic variability relating to the appearance and duration of each stage of development, due to a different sensitivity of the varieties to vernalization and photoperiod, and to an intrinsic earliness (Whitechurch et al., 2007). It is, therefore, possible that in these terms, relative to the appearance of the stem elongation phase, Mieti presented a difference with respect to the varieties such as San Giorgio, Mara, San Pastore, and Fortunato. As regards phenophases 40–49, 50–59, 61, and 89, no differences were observed for any of the varieties in the thermal thresholds necessary to reach them (*p*-value > 0.05). To summarize, differences between the two periods in chronological time (DAS) for achieving the phenological stages from booting forward were not due to a different precocity of the varieties but were the effect of the increase in temperatures produced by CC.

Starting from booting, and up to full ripening, a clear and significant (*p*-value < 0.05) anticipation of the wheat life cycle emerged in the present period, compared to the past period, on average, 169 ± 19 vs. 193 ± 8 days for booting, 174 ± 21 vs. 198 ± 7 days for heading, 178 ± 22 vs. 202 ± 7 days for beginning of anthesis, and 223 ± 22 vs. 244 ± 6 days for the achievement of stage 89. Furthermore, the high standard deviation in the present period compared to the past period from heading onward (about 3-folds) demonstrates the high inter-annual variability linked to CC. The phenological shortening due to CC rising temperatures is known to reduce both times for photosynthesis and assimilates translocation, hampering grain yield, as shown by the previous studies conducted in different parts of the world (Tao et al., 2008; Zacharias et al., 2010). This negative impact could be at least partially neutralized acting on crop calendar (e.g., sowing dates) and favoring crop cultivars with improved duration of delicate phenological stages, maximizing photosynthesis and translocation capacity, as also suggested by Fatima et al. (2020). However, this last point is controversial in the case of Mediterranean Europe, as bread wheat is traditionally cropped as a rainfed cereal in this area, typically experiencing drought stressing conditions from anthesis onward (Del Moral et al., 2003). So, short-cycle varieties have been widely selected in the area, to escape terminal drought (Shavrukov et al., 2017). A precise agronomic evaluation of the proper strategy to cope with the necessity of escaping terminal drought on one side, and of improving time for photosynthesis and resources allocation to the forming grain on the other, will be needed in the region.

The acceleration of grain filling, and the presence of frequent heat stress conditions during this stage under rising temperatures scenarios produced by CC, is well documented. Consequences on yield and kernel weight are significant (Djanaguiraman et al., 2020), being early grain filling associated with photosynthesis (van Dongen et al., 2004) and late grain filling with the remobilization of stored assimilates from vegetative tissues to the grain (Plaut et al., 2004). In our study, however, no differences in total amount of proteins were detected in seed samples derived from different years or periods, and kernels resulted in a mean protein content of 13.1% of total weight, in accordance with the values found in the literature for the San Pastore cultivar, i.e., 12.7 and 11.6% (Hristov et al., 2010; Horvat et al., 2021). The grain-filling stage might be crucial for the accumulation of gluten proteins, a complex process involving a finely tuned spatial and temporal regulation; so, the above-mentioned environmental changes might determine the changes also in gluten composition. The gluten fraction, i.e., gliadins and glutenins, constitutes the 80–85% of total grain proteins and contributes to rheological, pasting, and textural properties of dough (Shewry et al., 1995; Horvat et al., 2021). Hence, amount of protein, protein distribution among fractions, and the ratio between gliadins/glutenins and HMW-GS/LMW-GS in wheat flours derived from San Pastore kernels harvested in different years, from both before and after CC periods, were tested for correlation with CGDDs accumulated in the period 1 May–15 June, when wheat grain filling typically occurs in this area. Higher CGDDs accounted for both higher temperature in the period and fastened grain filling (typical consequence of CC in Southern Europe).

The HMW-GS and HMW-GS/LMW-GS ratio showed strong and significant correlation with 1 May–15 June CGDDs, suggesting that higher temperatures during the filling phase and its fastening lead to higher molecular weight glutenins. On the contrary, gliadins showed a significant negative correlation with CGDDs. Same trends have been recently reported for two bread wheat cultivars grown under moderate temperatures during grain filling (Koga et al., 2015). Koga et al. explored the effects of low to moderate temperatures on the proportions of gluten proteins and the assembly of glutenin polymers, using temperatures ranging from 13 to 23°C during the early period of grain filling. The authors thus did not subject plants to heat stress, which occurs with temperatures exceeding 30°C (Koga et al., 2015). Anyway, similar to the results herein reported, authors noted that the duration of grain filling was longer at the lower temperatures, and that temperature, despite having less effect on the amount of protein accumulated per grain, significantly altered the proportion of gluten proteins. Notably, the proportions of different gluten proteins, i.e., gliadins, HMW-GS, and HMW-GS/LMW-GS ratio, correlated with CGDDs for the San Pastore cultivar in a similar manner the temperature did for both investigated cultivars analyzed by Koga et al. (2015). The effects of air temperature on the grain filling of wheat seem evident, as it is the main environmental factor regulating phenological development. However, only a few studies have focused on understanding how various genotypes and environmental factors interact in the creation of the protein polymer structure that determines bread-making quality. In general, the higher the temperature, the faster is the rate of development and, consequently, the shorter is the time to complete each life cycle stage (Slafer et al., 2009), providing that temperature does not exceed the upper threshold. The reduction of the chronological time available for the grain-filling stage is of high importance in determining gluten protein polymer structure, even more than the specific protein composition (Malik et al., 2013), as confirmed by our case study.

Alterations in the composition of gluten may alter the properties of the dough, but the result remains speculative, as there are many factors involved in the formation of the protein network in the process of bread-making. In general, the good bread-making performance of a dough depends in fact on a delicate balance of elasticity and viscosity (He et al., 2005).

Summarizing, this case study assessed the impact of CC in the Bolognese Plain (North of Italy) and quantified its effect on bread wheat phenology, highlighting a significant shortening of the wheat life cycle. The new climate also accounted of a higher heat unit accumulation (CGDD) in the typical period of grain filling (1 May –15 June), meaning that wheat is subjected to frequent heat stress during this delicate period of grain protein accumulation, and to a faster process, reducing the time available for a proper grain filling. Analyzing grain samples from before and after the CC periods, this phenomenon showed a significant correlation with gluten protein composition, consistently altering wheat grain quality.

## Conclusion and future perspectives

Although it is well known that CC can cause a shift in crops’ phenological development, studies that exactly quantify this shift are few, due to the lack of long lasting phenological surveys, conducted in the same site and on the same varieties along an appreciable time span.

In this scenario, this case study precisely quantifies CC effects in the experimental site, providing very rigorous data, characterizing the area under examination in detail, coupling phenological and meteorological data collected in the same site, and on the same varieties, for two long and continuous periods of time, before and after CC.

In particular, this study precisely assesses the shortening of bread wheat phenological development, in a key area for its cultivation, such as Southern Europe. The acceleration of wheat life cycle in the CC scenario can have negative consequences on grain yield, due to the shortening of phenological phases, such as vegetative and grain-filling period, reducing the time available for both photosynthesis and assimilates translocation to the forming grains (Zacharias et al., 2010). At the same time, in the new conditions, the crop is more often subjected to heat stress in delicate phases, such as flowering, resulting in grain yield losses even higher than 50% (Nahar et al., 2010). Moreover, altering carbon balance and evapotranspiration demand can be handled only by means of smart management of the agro-ecosystem (Fatima et al., 2020).

So, this case study represents an interesting source of information for modeling purposes, aimed at elaborating climate smart agriculture strategies, to mitigate the impact of rising temperatures on wheat yield and grain quality, such as the change in cropping calendar (e.g., sowing date), the breeding of new cultivars with improved duration of critical phenological phases, the optimization of management practices (e.g., fertilizers and irrigation water use efficiency). The exact definition of crop growth phase alterations also provides clear indications of CC influence on biological processes at agro-ecosystem level in the area, being phenology the most common feature of crop adaptation to the climate (Fatima et al., 2020).

Moreover, as far as we know, we report for the first time on how gluten composition mirrors phenological alterations in Mediterranean climate by analyzing a historical seed collection of the same variety, cropped in the same study area. More of these types of studies would be desirable, but they are often not possible, given the limited availability of historical seed collections for destructive analysis. Instead, it is important to use seeds coming from experimental and certification activities carried out in the past, like Laras laboratory. This kind of collection might be used to deepen the studies on seed quality alterations, in support of plant breeding from the perspective of CC. This case study highlights how technological grain quality (i.e., gluten composition) is threatened in an area of considerable importance for this cereal, on which global food security largely depends. In conclusion, the information included in this study represents a tool for modeling, for both predictive purposes and decision supporting systems for farmers, as well as can guide future breeding choices for varietal innovation.

## Data availability statement

The original contributions presented in this study are included in the article/Supplementary material, further inquiries can be directed to the corresponding author.

## Author contributions

GP, FV, and IA planned the research and interpreted the data. GP, SC, and IA analyzed the data and prepared the figures. SC and SP provided the seed material and pheno-meteorological data. GP and SC performed the molecular analysis. MN and JF developed the protocol for gluten analysis. EE performed HPLC analysis. GP, IA, and FV wrote the article with contributions from all other authors. All authors contributed to the article and approved the submitted version.

## Footnotes

1
https://www.fao.org/faostat/en/#data/QCL

2
https://wheat.pw.usda.gov/GG3/

3
https://cran.r-project.org/bin/windows/base/old/
