# Supplementary material for: Climate change effects on bread wheat phenology and grain quality: A case study in the north of Italy
Source: Front Plant Sci. 2022 Aug 9;13:936991. doi: 10.3389/fpls.2022.936991 (PMC9396297; doi:10.3389/fpls.2022.936991)
Supplement: Supplementary file 1 [file Table_1.DOCX]

Supplementary Table 1_Complete list of the SSR (Simple Sequence Repeat) markers used to verify genotype homogenicity. The 19 Xgwm SSR markers were probed. SSR markers used were chosen randomly across the entire genome.

|  |  | 3B | 2A | 5B/5D | 5B/5D | 1A | 4A | 6B | 4B | 2A | 7B | 5B |
| --- | --- | --- | --- | --- | --- | --- | --- | --- | --- | --- | --- | --- |
| **Sample number** | **Year** | **Xgwm493** | **Xgwm304** | **Xgwm159.5D** | **Xgwm159.5B** | **Xgwm164** | **Xgwm165** | **Xgwm191** | **Xgwm192** | **Xgwm382** | **Xgwm577** | **Xgwm213** |
| **1** | 1955 | B | A | A | B | A | A | A | B | B | A | A/C |
| **2** | 1955 | B | A | A | B | A | A | A | B | B | A | A/C |
| **3** | 1961 | B | A | A | B | A | A | A | B | B | A | A/C |
| **4** | 1973 | B | A | A | B | A | ND | A | B | B | A | A/C |
| **5** | 2020 | B | A | A | B | A | A | A | B | B | A | A/C |
| **6** | 2016 | B | A | A | B | A | A | A | B | B | A | A/B/C |
|  |  |  |  |  |  |  |  |  |  |  |  |  |
|  |  | 6B | 3B/3A | 2A | 2A/4A | 2A/4A | 1B | 1B | 3B | 7D | 7B |  |
|  |  | **Xgwm219** | **Xgwm247** | **Xgwm249** | **Xgwm265.2A** | **Xgwm265.4A** | **Xgwm268** | **Xgwm274** | **Xgwm285** | **Xgwm295** | **Xgwm297** |  |
| **1** | 1955 | A | B | A/B | A/B | A/B | A/C | B | A | A | B |  |
| **2** | 1955 | A | B | A/B | A | A/B | A/C | B | A | A | B |  |
| **3** | 1961 | A | B | A/B | B | A/B | A/C | B | A | A | B |  |
| **4** | 1973 | A | B | A/B | A | A/B | A/C | B | A | A | B |  |
| **5** | 2020 | A | B | A | B | A/B | A/C | B | A | A | B |  |
| **6** | 2016 | A | B | A/B | A | A/B | A/C | B | A | A | B |  |
