# Supplementary material for: Climate change effects on bread wheat phenology and grain quality: A case study in the north of Italy
Source: Front Plant Sci. 2022 Aug 9;13:936991. doi: 10.3389/fpls.2022.936991 (PMC9396297; doi:10.3389/fpls.2022.936991)
Supplement: Supplementary file 2 [file Table_2.DOCX]

Supplementary Table 2_Complete list of identified proteins, divided in gliadins and glutenins.

| Alpha-gliadin OS=Triticum aestivum OX=4565 PE=4 SV=1 |
| --- |
| Gamma-gliadin (Fragment) OS=Triticum aestivum OX=4565 PE=4 SV=1 |
| CSB_alpha gliadin 7 OS=Triticum aestivum OX=4565 GN=Gli-2 PE=4 SV=1 |
| Omega-gliadin (Fragment) OS=Triticum aestivum OX=4565 GN=gli PE=2 SV=1 |
| Alpha-gliadin OS=Triticum aestivum OX=4565 GN=Gli-SB-3 PE=4 SV=1 |
| Gamma-gliadin OS=Triticum aestivum OX=4565 GN=ll926 PE=4 SV=1 |
| Alpha/beta-gliadin OS=Triticum aestivum OX=4565 GN=Gli-2 PE=4 SV=1 |
| Gamma-gliadin OS=Triticum aestivum OX=4565 PE=4 SV=1 |
| Gamma-gliadin OS=Triticum aestivum OX=4565 GN=ll934 PE=4 SV=1 |
| Alpha-gliadin OS=Triticum aestivum OX=4565 GN=gli-2 PE=4 SV=1 |
| Gamma-gliadin (Fragment) OS=Triticum aestivum OX=4565 PE=4 SV=1 |
| Alpha-gliadin storage protein OS=Triticum aestivum OX=4565 PE=4 SV=1 |
| Gamma-gliadin OS=Triticum aestivum OX=4565 GN=GID-HE1 PE=2 SV=1 |
| Alpha/beta-gliadin OS=Triticum aestivum OX=4565 PE=4 SV=1 |
| Alpha-gliadin OS=Triticum aestivum OX=4565 GN=gli-2 PE=4 SV=1 |
| Alpha/beta-gliadin OS=Triticum aestivum OX=4565 PE=4 SV=1 |
| Omega gliadin-B6 OS=Triticum aestivum OX=4565 GN=Gli-1 PE=4 SV=1 |
| Gamma-gliadin (Fragment) OS=Triticum aestivum OX=4565 PE=4 SV=1 |
| Alpha-gliadin OS=Triticum aestivum OX=4565 GN=Gli-Z3 PE=2 SV=1 |
| Alpha/beta-gliadin OS=Triticum aestivum OX=4565 GN=Gli-2 PE=4 SV=1 |
| Alpha-gliadin OS=Triticum aestivum OX=4565 GN=Gli-A2-5 PE=4 SV=1 |
| Gamma gliadin-A1 OS=Triticum aestivum OX=4565 GN=Gli-1 PE=2 SV=1 |
| Putative omega-gliadin (Fragment) OS=Triticum aestivum OX=4565 GN=gli PE=2 SV=2 |
| Gliadin/avenin-like seed protein OS=Triticum aestivum OX=4565 PE=2 SV=1 |
| Gliadin/avenin-like seed protein OS=Triticum aestivum OX=4565 PE=2 SV=1 |
| Alpha-gliadin OS=Triticum aestivum OX=4565 PE=4 SV=1 |
| Pseudo alpha/beta-gliadin OS=Triticum aestivum OX=4565 PE=4 SV=1 |
| Alpha-gliadin (Fragment) OS=Triticum aestivum OX=4565 GN=gli PE=2 SV=1 |
| Omega-gliadin (Fragment) OS=Triticum aestivum OX=4565 PE=4 SV=1 |
| Gamma-gliadin OS=Triticum aestivum OX=4565 PE=4 SV=1 |
| Gamma-gliadin OS=Triticum aestivum OX=4565 PE=4 SV=1 |
| Gamma-gliadin (Fragment) OS=Triticum aestivum OX=4565 PE=4 SV=1 |
| Gamma gliadin-B6 OS=Triticum aestivum OX=4565 GN=Gli-1 PE=2 SV=1 |
| Gamma gliadin-B4 OS=Triticum aestivum OX=4565 GN=Gli-1 PE=4 SV=1 |
| Alpha-gliadin OS=Triticum aestivum OX=4565 GN=Gli-6 PE=4 SV=1 |
| CSB_alpha gliadin 9 OS=Triticum aestivum OX=4565 GN=Gli-2 PE=4 SV=1 |
| Gamma-gliadin OS=Triticum aestivum OX=4565 PE=4 SV=1 |
| Alpha-gliadin OS=Triticum aestivum OX=4565 GN=Gli-CS-1 PE=4 SV=1 |
| Alpha-gliadin OS=Triticum aestivum OX=4565 GN=gli-2 PE=4 SV=1 |
| Alpha-gliadin OS=Triticum aestivum OX=4565 GN=Gli-B2-8 PE=4 SV=1 |
| Alpha-gliadin OS=Triticum aestivum OX=4565 GN=Gli-D2-13 PE=4 SV=1 |
| Gamma gliadin OS=Triticum aestivum OX=4565 PE=4 SV=1 |
| Alpha/beta-gliadin OS=Triticum aestivum OX=4565 GN=Gli-2 PE=4 SV=1 |
| Gamma-gliadin OS=Triticum aestivum OX=4565 PE=4 SV=1 |
| Gamma-gliadin OS=Triticum aestivum OX=4565 PE=4 SV=1 |
| Alpha-gliadin OS=Triticum aestivum OX=4565 GN=gli-2 PE=4 SV=1 |
|  |
|  |
| High-molecular-weight glutenin subunit 2.6 OS=Triticum aestivum OX=4565 GN=Glu-D1 PE=3 SV=2 |
| High molecular weight glutenin subunit 1Bx protein OS=Triticum aestivum OX=4565 GN=Glu-B1-1 PE=3 SV=1 |
| High molecular weight glutenin subunit 1Ax1 OS=Triticum aestivum OX=4565 GN=Glu-1Ax1 PE=3 SV=1 |
| High molecular weight glutenin subunit 1By15 OS=Triticum aestivum OX=4565 PE=2 SV=1 |
| Low-molecular-weight glutenin subunit (Fragment) OS=Triticum aestivum OX=4565 GN=LMW-GS PE=4 SV=1 |
| Low-molecular-weight glutenin subunit OS=Triticum aestivum OX=4565 GN=LMW-GS PE=4 SV=1 |
| Low-molecular-weight glutenin subunit Glu-B3 (Fragment) OS=Triticum aestivum OX=4565 PE=4 SV=1 |
| LMW-D1 OS=Triticum aestivum OX=4565 GN=GluD3-3 PE=2 SV=1 |
| Low-molecular-weight glutenin subunit OS=Triticum aestivum OX=4565 GN=LMW-GS PE=4 SV=1 |
| HMW glutenin subunit OS=Triticum aestivum OX=4565 GN=Glu PE=2 SV=1 |
| D-type LMW glutenin subunit (Fragment) OS=Triticum aestivum OX=4565 GN=glu PE=2 SV=1 |
| LMW-m glutenin subunit 47 (Fragment) OS=Triticum aestivum OX=4565 GN=LMW-m PE=4 SV=1 |
| Glu-B1-1b HMW glutenin subunit OS=Triticum aestivum OX=4565 PE=3 SV=1 |
| Low-molecular-weight glutenin subunit OS=Triticum aestivum OX=4565 GN=LMW-GS PE=4 SV=1 |
| Low molecular weight glutenin subunit LMW-9 OS=Triticum aestivum OX=4565 PE=4 SV=1 |
| Glutenin, low molecular weight subunit PTDUCD1 OS=Triticum aestivum OX=4565 PE=1 SV=1 |
| LMW-GS OS=Triticum aestivum OX=4565 GN=Glu-A3 PE=2 SV=1 |
| Low-molecular-weight glutenin subunit (Fragment) OS=Triticum aestivum OX=4565 GN=LMW-GS PE=4 SV=1 |
| HMW glutenin subunit Ax2 OS=Triticum aestivum OX=4565 GN=HMWG PE=3 SV=1 |
| Low molecular weight glutenin subunit OS=Triticum aestivum OX=4565 GN=GluB3-2 PE=4 SV=1 |
| Low molecular weight glutenin subunit OS=Triticum aestivum OX=4565 GN=GluB3-3 PE=4 SV=1 |
| Low molecular weight glutenin subunit OS=Triticum aestivum OX=4565 GN=GluB3-4 PE=4 SV=1 |
