# Supplementary figures and images for: Climate change effects on bread wheat phenology and grain quality: A case study in the north of Italy
Source: Front Plant Sci. 2022 Aug 9;13:936991. doi: 10.3389/fpls.2022.936991 (PMC9396297; doi:10.3389/fpls.2022.936991)

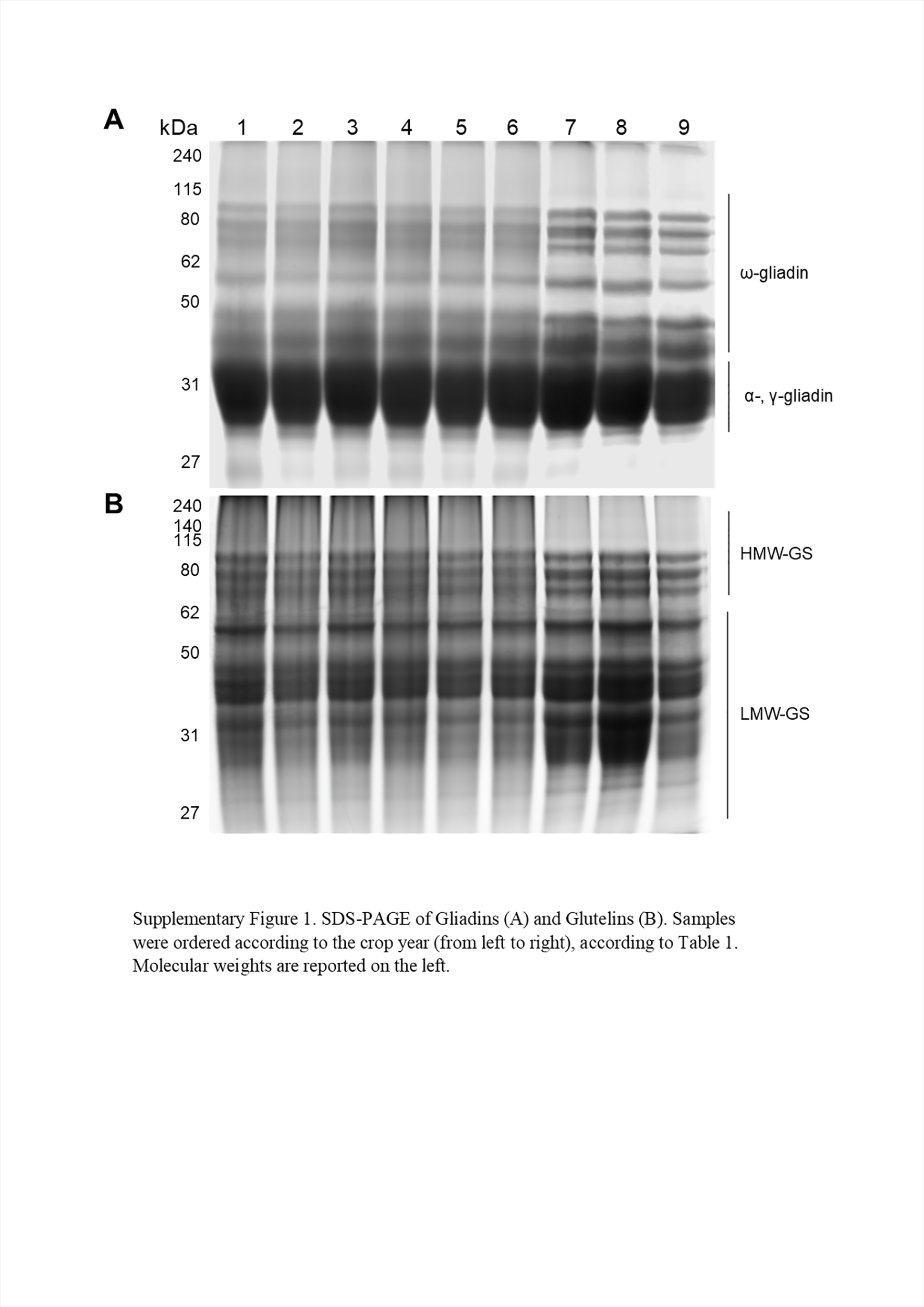

Supplement: Supplementary file 3 [file Image_1.TIF]
